# Purification of Stem Cells from Oral Pyogenic Granuloma Tissue

**DOI:** 10.2174/1874210601812010560

**Published:** 2018-08-29

**Authors:** Ali Dehghani Nazhvani, Shamsedin Ahzan, Seyed-Mojtaba Hosseini, Armin Attar, Ahmad Monabati, Maryam Sadat Tavangar

**Affiliations:** 1 Department of Oral and Maxillofacial Pathology, School of Dentistry, Shiraz University of Medical Sciences, Shiraz, Iran; 2 Biomaterials Research Center, School of Dentistry, Shiraz University of Medical Sciences, Shiraz, Iran; 3 Students' Research Committee, Cellular and Molecular Research Club, Shiraz University of Medical Sciences, Shiraz, Iran; 4 Cellular and Molecular Research Club, Shiraz University of Medical Sciences, Shiraz, Iran; 5 Cardiovascular Research Center, Shiraz University of Medical Sciences, Shiraz, Iran; 6 Hematology Research Center, Shiraz University of Medical Sciences, Shiraz, Iran; 7 Molecular Pathology Research Center, Shiraz University of Medical Sciences, Shiraz, Iran; 8 Department of Operative Dentistry, School of Dentistry, Shiraz University of Medical Sciences, Shiraz, Iran

**Keywords:** Adult stem cell, Pyogenic granuloma, Stem cell assay, Reactive oral lesion, Osteoblasts, Mesenchymal stromal cells

## Abstract

**Introduction:**

The isolation of stem cells from pathologically damaged dental tissues has been examined only by a few studies. The purpose of this study was to evaluate the possibility of isolation of stem cells from pyogenic granuloma.

**Methods:**

Pyogenic granuloma tissues were enzymatically digested and the resulting single cells were cultured. Then, the cultured cells differentiated into adipocytes and osteoblasts cells. Flow cytometry analyses were performed on markers such as CD 90, CD 73, CD105, CD 45 and CD14. Other features were also analyzed including the effect of colony formation and potentials of differentiation into adipocytes and osteoblasts.

**Results:**

The cells derived from pyogenic granuloma tissue formed higher colonies similar to typical spindle-shaped fibroblasts. The cells were positive for mesenchymal markers such as CD 44, CD 271, CD 90, and CD 73, and negative for surface molecules such as CD 14, CD 34 and CD 45. Moreover, they successfully differentiated into adipocytes and osteoblasts.

**Conclusion:**

The cells isolated from pyogenic granuloma could form CFU fibroblastic units expressing an appropriate marker panel of the cell surface antigen and adequate differentiation potential, all of which met the Cell Therapy International Association criteria for the definition of mesenchymal stromal cells. Pyogenic granuloma contains cells with stem cell properties.

## 1 INTRODUCTION

Stem cells are the main sources of cells needed to reconstruct and regenerate the tissue. They are isolated from almost any type of tissue such as skin, brain, myocardial tissue, bone marrow, and dental pulp [1, 2]. Some of them have shown promising potentials for *in-vitro* expansion [3]; while, others have been proven to have no potential [4]. Their ability of self-renewal and differentiation is influenced by many factors like disease conditions, age, and exercising [5].

Multipotent Mesenchymal Stromal Cells (MSCs), previously known as “mesenchymal stem cells”, are the plastic adherent cells that are capable of multiple differentiation into mesenchymal and/or non-mesenchymal lineages such as hepatocytes, osteoblasts, cardiac cells, and adipocytes [6]. Conventionally, the MSCs are isolated from the bone marrow; although they can be obtained from other tissues such as umbilical cord blood [7, 8], adipose tissue [9], granulocyte-colony stimulating factor-mobilized peripheral blood [10], and Dental Pulp (DPSCs) [11]. Human dental pulp and its adjacent tissue are derived from ectomesenchyme; *i.e*., the tissues derived from interactions of neural crest cells and mesenchymal tissues during embryogenesis. Recently, ectomesenchymal dental tissues such as periodontal ligament, have been widely studied for stem cells [12]; but unfortunately, only a few studies have evaluated the pathologically damaged tissues regarding the presence of stem cells [13-15].

Pyogenic granuloma is a common reactive lesion of the oral cavity with up to 85% predilection for the gingiva [16]. It is treated through excisional surgery and is believed to be a good reservoir of applicable stem cells [17]. This study tried to evaluate the possibility of isolating stem cells from pyogenic granuloma.

## 2 MATERIALS AND METHODS

### 2.1 Preparation of Single-Cell Suspension from Pyogenic Granuloma

**Fig. (1):**
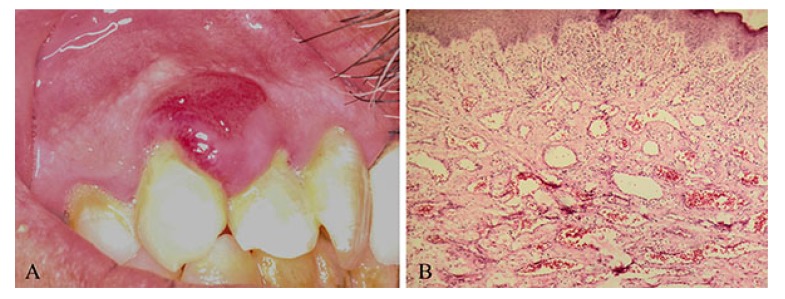
Morphological and histological views of Pyogenic granuloma. **A)** Clinical view of the pyogenic granuloma placed on upper attached gingiva between lateral and canine teeth. **B)** Section of the pyogenic granuloma excised out of one patient. Note the oral epithelium and the granulation tissue beneath (H&E staining, X200).

Four samples of pyogenic granuloma were collected from the patients referring to Shiraz School of Dentistry. All samples were located on gingiva, and an experienced pathologist confirmed the diagnosis through pathological sections (Fig. **1**). The patients signed an informed consent before being enrolled in the study. The study was in accordance with the Declaration of Helsinki and was approved by the local ethics committee.

The samples of pyogenic granuloma tissue were transferred to PBS-EDTA solution with 1% penicillin/streptomycin and 1% fungizone (both from Gibco/Invitrogen Company, Carlsbad, California, USA). The tissues were broken under sterile conditions by using enzymatic digestion while occasionally shaking for 1 hour in a solution of 3 mg/mL collagenase type 1, and 4 mg/mL dispase type 2 (both from Sigma, St. Louis, Missouri, USA). The resulting single-cell suspension was passed through a 70-micron cell filter (Biosciences BD, San Jose, California, USA) and centrifuged for 10 minutes to remove the supernatant remaining enzymes. The cells were re-suspended in the environment and each sample was separately used for the next steps including evaluation of the potential of colonization or cultivation for further analysis.

### 2.2 Isolated Cells Culture

The single-cell suspensions were seeded onto cultivation flasks within the α-MEM along with 4 mM GlutaMax, 100 U/ml penicillin, 100 mcg/ml streptomycin and 20% FBS (all from Gibco/Invitrogen). The cells were cultured for 72 hours at 37°C in 5% CO_2_ and 90% humidity. Then, the free cells and residuals were removed and fresh medium was added to the adherent cells. The medium was changed twice a week until the flask reached 80% confluence. The cells were then released with Trypsin- EDTA (Gibco/Invitrogen) and the second culture was performed. They were passaged three times before they could be used for differentiation analysis or flow cytometry.

### 2.3 Colony Forming Unit Assay

To evaluate the efficiency of the derived single-cell colonization (colony-forming unit fibroblast assay [CFU-F]), the primary cells derived from the pyogenic granuloma were cultured on six-chamber plates with the living cell concentration of 1000 cells per ml from the culture medium. The single-cell derived colonies were defined as those units with more than 50 cells.

### 2.4 Adipogenic Differentiation

As formerly mentioned, the third passage cells were collected for adipogenic differentiation and cultured in MesenCult medium along with 10% adipogenic stimulatory supplements (both from STEMCELL Technologies Inc., Vancouver, British Columbia, Canada) with a density of 2×10^3^ cells in a milliliter of medium within the two-chamber cultivation slides according to the manufacturer's instructions. The cells were cultured for 3-4 weeks. Half of the media were changed only when the ambient color turned yellow. When the cells exhibited appropriate morphological changes, they were analyzed by using Oil Red staining (Sigma). In order to perform the staining, the cells were incubated in 4% formalin containing 1% calcium chloride for one hour. The cells were then stained with Oil Red O solution for 10-15 minutes, and in ethanol 70% for one minute; and then, washed with distilled water.

### 2.5 Osteogenic Differentiation

For osteogenic differentiation, the third passage cells were collected and cultured in NH OsteoDiff medium (Miltenyi Biotec GmbH, Bergisch Gladbach, Germany) with a density of 10^4^×3 cells in a milliliter of the medium according to the manufacturer's instructions. The medium was replaced twice a week for three weeks. To verify the differentiation, after suitable morphological changes, the cells were washed with PBS and incubated in methanol for 10 minutes. They were stained with 1.0 M solution of red Alizarin in 25% ammonia water for 24 hours and washed with distilled water.

### 2.6 Flow Cytometry

Analysis of the cell surface antigen scenarios was done by using the cells obtained from the third passage. The isolated cells were incubated for 10-30 minutes in a dark environment with the following anti-human antibodies: CD90- fluorescein isothiocianate (FITC), CD 34- phycoeritrin (PE), CD 45- Peridinin chlorophyll protein (PerCP), CD 271-FITC (MiltenyiBiotec), CD106-PE, CD 44-FITC, CD73-PE, CD14-FITC (BD Biosciences), and CD105-PerCP (AbDSerotec, Kidlington, Oxford, England). Isotype-matched irrelevant monoclonal antibodies such as Mouse IgG1-PE, IgG2a-FITC (AbDSerotec, Kidlington, Oxford, England), IgG2a-PerCP, IgG2b-FITC and IgG2a-PE (Miltenyi Biotec) were used to omit the effect of non-specific cells staining. Flow cytometry analysis was done in a FACS Calibur device (BD Biosciences) by using the cell quest as data acquisition software. Win MDI software, version 8.2, was employed for data analysis.

## 3 RESULTS

### 3.1 Culture Specifications

**Fig. (2):**
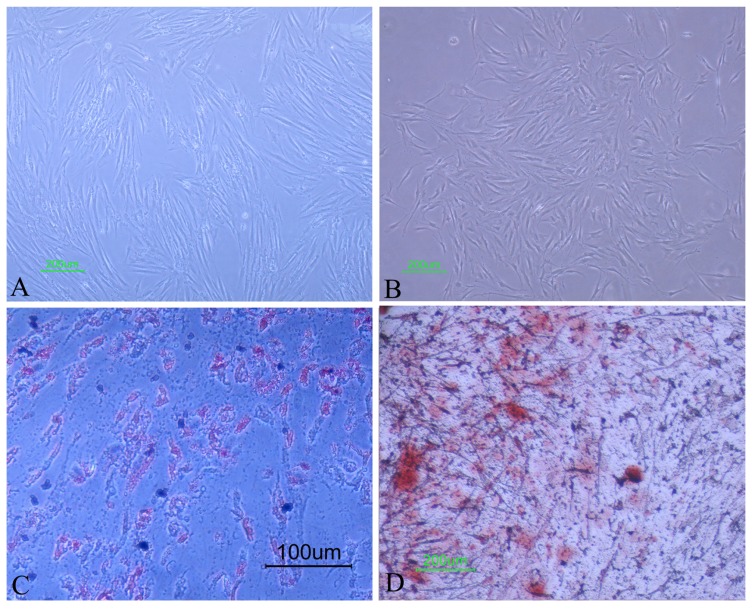
Pyogenic granuloma-derived cells. **A)** After the initial seeding, many cells attached to the plates in an acceptable fibroblast-like (fusiform) appearance. These cells reached confluence within 12-21 days. **B)** A single cell derived colony from pyogenic granuloma-derived cells formed within the CFU-Fibroblast assay. **C)** Lipid vacuoles stained with Oil Red O stain after adipogenic differentiation. **D)** Osteogenic differentiation with calcium deposition identified by Alizarin red staining. (*Abbreviations: CFU-F = Colony forming unit fibroblast)*.

The pyogenic granuloma-derived cells attached to plates two to five days after the primary culture. These cells reached confluence in 12-21 days, with a proper appearance similar to spindle-shaped fibroblast (Fig. **2A**). They could also successfully form single-cell derived colonies (Fig. **2B**).

### 3.2 Colony Forming Unit Assay

To assess single cell-derived colony formation (Colony Forming Unit-Fibroblasts [CFU-F assay]), only colonies with more than 50 cells were considered in colony enumeration (Fig. **2B**). From 1000 plated pyogenic granuloma tissue-derived cells 11.3 ± 8.2 colonies were formed.

### 3.3 Differentiation Analysis

Adipogenic differentiation was confirmed through morphological changes and the corresponding staining. One week after cultivation in adipogenic medium, the cells showed small isolated vacuoles whose number and size increased over time, and all of them were stained with Oil Red O (Fig. **2C**). The earliest evidence of differentiation into osteoblasts matrix sediments was observed during the second week. Full differentiation into osteoblast cells lasted four weeks. Alizarin red staining documented the mineralization (Fig. **2D**).

### 3.4 Flow Cytometry Results

Analysis of the flow cytometry of the cells obtained from cultures of pyogenic granuloma-derived cells showed that the cells were positive for mesenchymal markers such as CD 44, CD 271, CD 90, CD 73, and negative for the surface molecules like CD 14, CD 34 and CD 45. These show a heterogeneous phenotype for CD105 and CD106 (data from one of the samples are shown in Fig. (**3**)).

## 4 DISCUSSION

This study evaluated the possibility of stem cell purification from samples of soft tissues pyogenic granuloma of the oral cavity. These tissues were found to contain cells with properties of stem cells.

At the time, dental pulp stem cells are the main source of cells for the regeneration of dental tissues. However, they are only accessible through extraction of a tooth with intact and natural pulp. As a result, they are not always considered practical and viable options. Alternatively, the stem cells can be extracted from oral pathologic lesions. Earlier, Wang *et al*. showed that the pulps damaged by irreversible pulpitis are likely to contain potential stem cells [18]. The same was shown in inflamed periapical tissue [19] and inflamed periodontal ligament tissue [20]. Working on dental pulp polyps was the first attempt to show the possibility of purification of dental pulp stem cells out of a dental pathologic lesion [13]. The current study revealed that other reactive oral lesions like pyogenic granuloma, also contain cells with stemness properties (ability of self-renewal and differentiation). The cells isolated from pyogenic granuloma could form CFU fibroblastic units with panels of the cell surface antigen marker and appropriate differentiation potential, all of which would meet the criteria of the International Society for Cellular Therapy for the definition of MSC [21].

Among the limitations of clinical use of the stem cells derived from pyogenic granuloma is the increased risk of contamination as the result of being exposed to the contents of oral cavity. To overcome this limitation in this study, antibacterial and antifungal supplements were employed from the very moment of tissue collection up to the final stages of sample preparation.

## CONCLUSION

Based on the findings of this study, it can be concluded that pyogenic granuloma contains cells with the stem cell properties. Further studies in this area are recommended to investigate more practical details about the possibility of clinical use of stem cells isolated from pyogenic granulomas.

## Figures and Tables

**Fig. (3):**
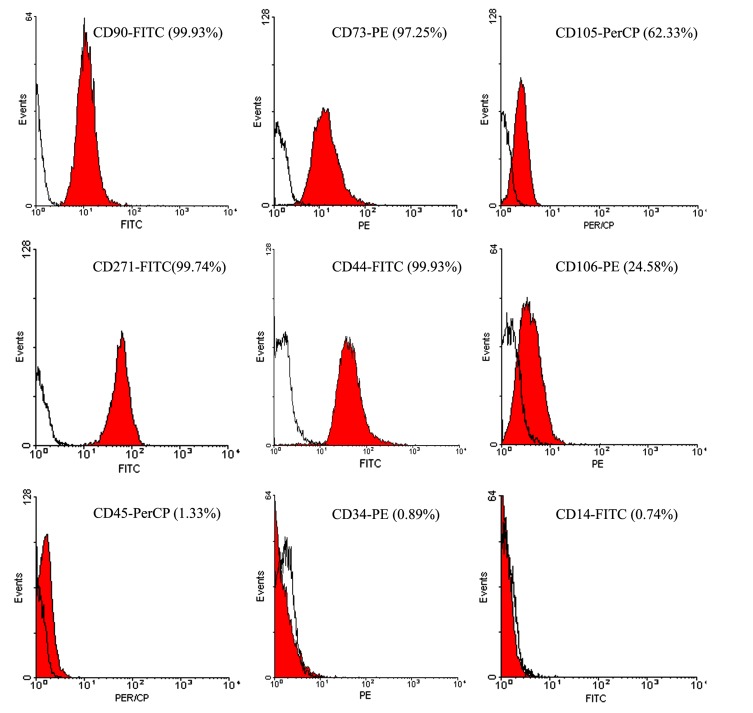
Flow-cytometric analysis of the pyogenic granuloma-derived cells in-vitro revealed a high level expression of CD 44, CD 271, CD 90, and CD 73 accompanied by low frequency for surface antigens: CD 14, CD 34 and CD 45. But these cells showed a heterogeneous phenotype for CD 105 and CD 106.

## LIST OF ABBREVIATIONS

APC: Allophycocyanin,
BM: Bone Marrow,
CFU-F: Colony Forming Unit Fibroblast,
DPSC: Dental Pulp Stem Cell,
FITC: Fluorescein Isothiocyanate,
MNC: Mononuclear Cells,
MSC: Multipotent Mesenchymal Stromal Cell,
PE: Phycoerythrin,
PerCP: Peridinin Chlorophyll Protein,
